# Cervical cancer screening delay and associated factors among women with HIV in Lesotho: a mixed-methods study

**DOI:** 10.1186/s12905-024-03382-8

**Published:** 2024-10-01

**Authors:** Michelle T. Harder, Moliehi Mokete, Frédérique Chammartin, Malebanye Lerotholi, Lipontso Motaboli, Mathebe Kopo, Mpho Kao, Moleboheng Mokebe, Ntoiseng Chejane, Palesa Mahlatsi, Morongoe Nyakane, Tapiwa Tarumbiswa, Niklaus D. Labhardt, Nadine Tschumi, Jennifer M. Belus

**Affiliations:** 1 Division of Clinical Epidemiology, Department of Clinical Research, University Hospital Basel, Totengässlein 3, Basel, 4051 Switzerland; 2 University of Basel, Basel, Switzerland; 3 SolidarMed, Partnerships for Health, Maseru, Lesotho; 4 Ministry of Health Lesotho, Maseru, Lesotho

**Keywords:** Cervical cancer screening, Access to care, HIV, Africa, Lesotho, Mixed-methods, Qualitative research

## Abstract

**Background:**

Cervical cancer is the fourth most common cancer in women worldwide, and women with human immunodeficiency virus (HIV) are particularly at risk of developing it. Regular screening effectively prevents morbidity and mortality. This mixed-methods study quantitatively assessed cervical cancer screening uptake and qualitatively explored the process of undergoing cervical cancer screening to understand possible reasons for delayed screening among women with HIV in Lesotho.

**Methods:**

Between October 2020 and March 2022, the *Vi*ral load *T*riggered *A*RT care in *L*esotho (VITAL) trial enrolled women aged 18 years and older with HIV who were taking antiretroviral therapy (ART). Cervical cancer screening delay was defined as reporting a screening that occurred more than two years ago or never having been screened. Cervical cancer screening uptake and the association between screening delay and sociodemographic variables were assessed using a multivariable mixed-effects logistic regression model accounting for clustering at clinic level. In-depth interviews were conducted with 16 women to obtain information on awareness, perceptions, and barriers to cervical cancer screening and were analyzed using thematic analysis.

**Results:**

Quantitative data were available for 3790 women. Among them, cervical cancer screening was delayed in 1814 (47.9%), including 1533 (40.5%) who were never screened. Compared to women aged 25 to 39 years, women aged 18 to 24 years (adjusted odds ratio (aOR) 2.8; 95% confidence interval (CI) 2.1–3.7), women aged 40 to 59 years (aOR 1.3; CI 1.1–1.6), and women older than 60 years (aOR 3.9; CI 3.0-5.1) were at higher risk of screening delay. Furthermore, time on ART below 6 months (aOR 1.6; CI 1.1–2.3) compared to above 6 months was associated with screening delay. Qualitative data identified limited awareness of cervical cancer risks and screening guidelines, misconceptions and fears created by the influence of other women’s narratives, and low internal motivation as the main barriers to screening uptake.

**Conclusions:**

Cervical cancer screening delay was common. Limited personal awareness and motivation as well as the negative influence of other women were the primary internal barriers to cervical cancer screening. Awareness and screening campaigns in Lesotho should consider these factors.

**Trial registration:**

clinicaltrials.gov, NCT04527874, August 27, 2020.

**Supplementary Information:**

The online version contains supplementary material available at 10.1186/s12905-024-03382-8.

## Background

Cervical cancer is the fourth most common cancer in women worldwide [1]. In 2020, more than 600,000 women developed cervical cancer and around 340,000 women died from it globally [1]. Approximately 84% of all cervical cancers, and nearly 9 out of 10 cervical cancer deaths, occur in low-income countries [2]. Cervical cancer is the leading cause of cancer-related deaths in women in eastern, western, middle and southern Africa [2].

The high burden of cervical cancer in low-income countries is due to various factors, including limited access to effective prevention strategies and high prevalence of carcinogenic human papillomavirus (HPV) strains and HIV [3, 4]. While HPV infection is the primary cause of the development of cervical cancer, HIV-related immunodeficiency significantly increases the risk of developing the disease [4]. Women with HIV have a six-fold higher risk of developing cervical cancer compared to women who do not have HIV [3]. The presence of HIV increases the risk of developing cancer due to higher incidence and reduced clearance of HPV [5, 6]. HPV vaccination and regular cervical cancer screening to detect HPV infection, as well as precancerous or early cancerous lesions, are effective tools to prevent morbidity and mortality from this disease [7, 8]. In southern Africa, where HIV prevalence is the highest in the world [4], scaling up cervical cancer screening has become a priority [9].

Pooled estimates from meta-analyses and demographic health surveys report cervical cancer screening coverage among women in African countries to be between 13% and 19%, with considerable variation between countries and sociodemographic subgroups [10, 11]. Studies among women with HIV in Ethiopia [12], South Africa [13], and Uganda [14] report cervical cancer screening uptake of 24.8%, 39.2% and 30.3%, respectively. Younger age, irregular income, lower level of education, and being unemployed were associated with low screening coverage [12, 15]. Furthermore, research suggests that barriers to screening service uptake among women with HIV are mainly due to limited knowledge and awareness as well as fear of cervical cancer screening and embarrassment, especially when screened by a male healthcare provider [16, 17]. Long waiting times, distance, and difficult-to-reachlocations of the healthcare facilities provide further barriers, suggesting that many factors potentially play a role in cervical cancer screening uptake.

Therefore, given the high risk of cervical cancer in women with HIV and limited research done on this topic in Lesotho, we conducted a study to understand (1) cervical cancer screening coverage and (2) reasons for delayed screening among women attending HIV care in Lesotho.

## Methods

### Study design and setting

This was a mixed-methods study consisting of both quantitative and qualitative components. For the comprehensive and transparent presentation of our research findings, we adhere to the Journal Article Reporting Standards (JARS) guidelines [18]. This study was nested within a larger clinical trial, *VI*ral load *T*riggered *A*RT care in *L*esotho (VITAL, NCT04527874), which is a multi-center cluster-randomized trial assessing the effect of an automated differentiated service delivery model on the clinical outcomes of people with HIV. The VITAL trial enrolled adults taking ART at 18 rural, nurse-led health clinics between October 2020 and March 2022 in the districts of Butha-Buthe and Mokhotlong in Lesotho, and builds on the Viral Load Cohort North-East Lesotho (VICONEL) [19]. Details on the VITAL trial have been published elsewhere [20].

Lesotho is a small mountainous country, landlocked by South Africa [21]. Nearly 50% of the approximately 2.2 million people live below the international standards for poverty, and 70% live in rural areas [22]. The HIV prevalence among women aged 15 to 49 years is 23.5% [23]. Cervical cancer is the most common cancer among women in Lesotho [24], and 57.9% of cervical cancers are attributable to HIV [3]. In 2013, Lesotho launched a cervical cancer screening program [25]. National guidelines for cervical precancer screening in Lesotho recommend screening for women with HIV every two years, starting at age 25 or when becoming sexually active [26]. The HPV vaccine was reintroduced in 2022 [27], and is recommended for girls aged 9–14 and girls and women living with HIV [26].

### Participants and research team

Participants were women living with HIV aged 18 years and older, taking ART, and registered for HIV care at one of the study’s clinics from the VITAL trial [20].

The research team was composed of Masters students as well as Bachelors-, Masters- and PhD-level academics from Lesotho, Switzerland, and Canada and had diverse professional backgrounds in medicine, psychology, epidemiology, social sciences, and statistics. The team members identified as cisgender women and men, ranging from their mid-20s to late 40s, and identified as belonging to Black African and White racial groups. The research members who conducted the interviews were local expert nurses who received training in conducting qualitative interviews.

### Procedures

Quantitative data were obtained during the baseline assessment of participants enrolled in the VITAL trial, where data on socio-demographic (age in years, occupation, education level, relationship status) and clinical characteristics (contraception type and reliability, pregnancy and breastfeeding status) were collected or obtained through the underlying VICONEL cohort database (baseline viral load, duration on ART). Participants also self-reported the time traveled to the health facility and their most recent cervical cancer screening date. Delay in screening was defined according to the Lesotho guidelines for HIV prevention [26] implemented during the study period as either never having been screened or a screening that occurred more than two years prior to the study interview date.

Qualitative data were collected from a convenience sample of VITAL participants who returned to two selected clinics (one per study district) between February and June 2022. These participants were invited to take part in a qualitative sub-study that provided feedback on the VITAL intervention and general healthcare engagement, including cervical cancer screening. Participants had to agree to have the interview audio-recorded to be eligible. There were no further eligibility criteria for the qualitative sub-study enrolment. Trained research assistants conducted individual in-depth interviews in Sesotho, the local language, using a specifically developed semi-structured interview guide (see supplementary material). The final interview guide underwent a forward- and back-translation process between English and Sesotho to ensure the meaning of questions was retained. Questions in the guide focused on women’s awareness, perceptions, and experiences related to cervical cancer screening. A professional bilingual local translator transcribed and translated all transcripts into English. The data collection process was overseen by a member of the research team.

### Ethics statement

The VITAL study protocol, including the nested qualitative study, has been approved by the National Health Research Ethics Committee (NH-REC) in Lesotho (ID 220–2019). All participants provided written informed consent for the VITAL trial and the nested qualitative study. Participants who were illiterate provided informed consent with a thumbprint, which was observed and signed by a witness. Participants received no remuneration for any component of study participation.

### Data analysis

For the quantitative analyses, we assessed the effect of participant characteristics on cervical cancer screening delay (i.e., never screened or most recent screening more than two years ago) using a multivariable mixed-effects logistic regression model accounting for clustering at the clinic level. Predictor variables were age, education level, more recent viral load (within 2 years prior to enrolment), employment status, relationship status, whether pregnant or breastfeeding, time on ART, travel time to the clinic, contraception type, and contraception reliability. We first ran univariable models and maintained all predictor variables with a p-value below 0.2 for the multivariable model. Contraception type and contraception reliability were removed from the multivariable model due to collinearity with age.

For the qualitative analyses, two members of the research team read the translated transcripts. During the familiarization process, they reviewed the first seven transcripts and noted initial open codes related to cervical cancer and its screening using an inductive approach. These open codes were used to create a codebook that included identified codes, definitions, and example quotes. Afterwards, the transcripts were systematically coded using the codebook, with the possibility that new codes could emerge during the coding process. Two coders independently coded all transcripts, and any discrepancies were reviewed and discussed. If the coders were not able to resolve the discrepancies, the last author served as a tiebreaker.

The finalized coded transcripts were used to conduct thematic analysis. Text segments labelled with the same codes were reviewed for patterns and themes, which included examining the intersection with other codes. Codes were then combined to form themes. The interrelationships between themes were examined and expressed diagrammatically. Microsoft Word was used to code transcripts and organize the coded data.

## Results

### Quantitative findings

Among 5801 VITAL trial participants with available baseline data, 3824 were female. We excluded 34 participants who did not answer the cervical cancer screening questionnaire, resulting in a final sample size of 3790 participants for the current analysis. Cervical cancer screening within the last two years was reported by 52.1% (*n* = 1976); 7.4% (*n* = 281) reported screening more than two years ago; and 40.5% (*n* = 1533) reported never having been screened.

**Fig. 1:**
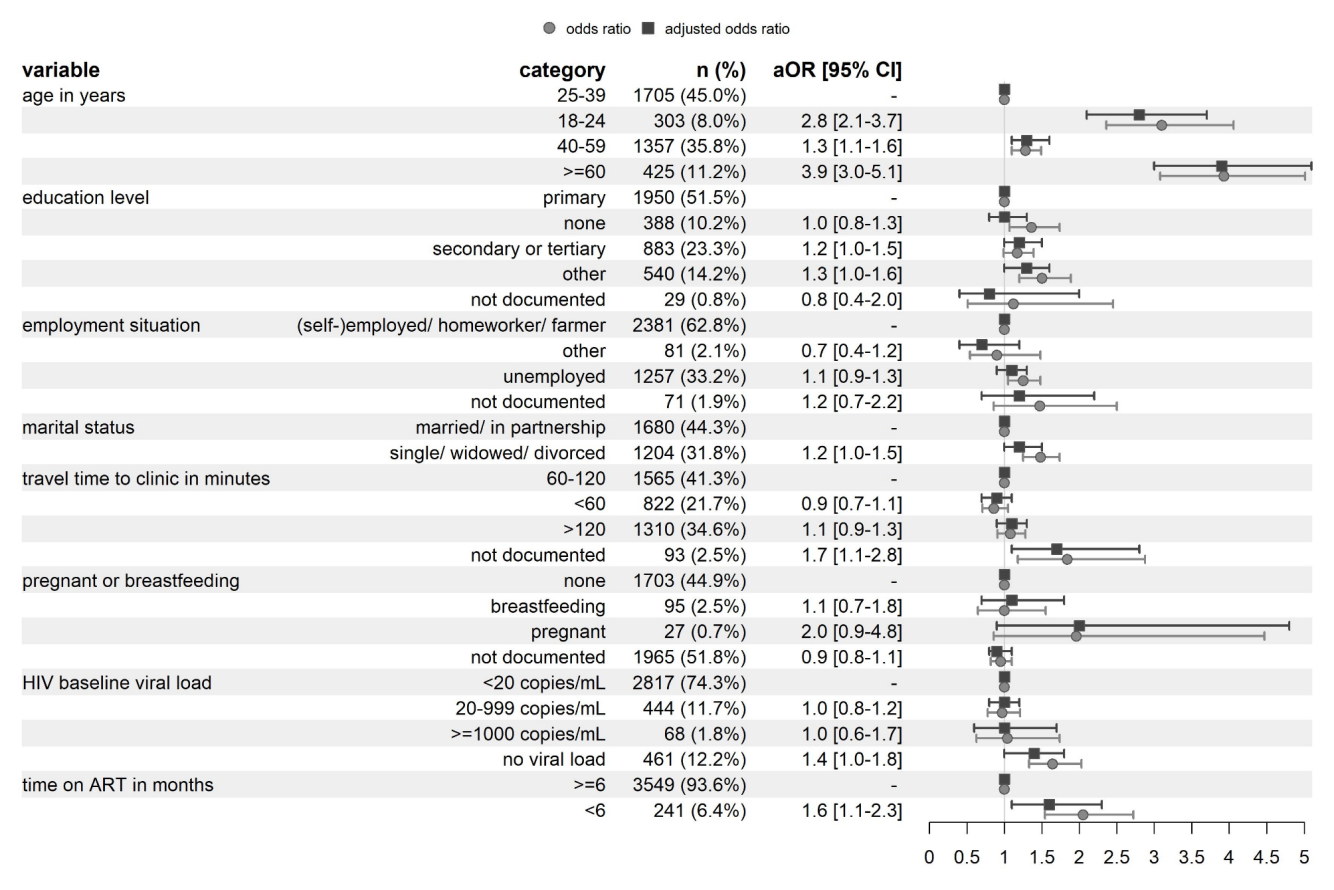
Factors associated with cervical cancer screening delay assessed through a multivariable mixed-effects logistic regression model among 3790 women. aOR: adjusted odds ratio; ART: antiretroviral therapy; CI: confidence interval. Note: All models include random effects at the level of the clinic. The vertical line on the forest plot at 1 indicates equality of odds between two given covariates categories, keeping all other covariates fixed

Participant characteristics and results of the multivariable mixed-effects logistic regression model for cervical cancer screening delay are provided in Fig. 1. Most participants were either (self-)employed, homeworkers (a homeworker was defined as someone doing private, unpaid care work and managing the household), or subsistence farmers (62.8%; *n* = 2381), had completed primary education (51.5%; *n* = 1950), were between 25 and 39 years old (45.0%; *n* = 1705), and were married or in a partnership (44.3%; *n* = 1680). Most participants had been taking ART for six months or longer (93.6%; *n* = 3549) and had a suppressed viral load measurement within 2 years prior to VITAL enrolment (74.3%; *n* = 2817). The multivariable logistic regression showed an increased adjusted odds ratio (aOR) for cervical cancer screening delay for the variables age, time on ART, and travel time to the clinic. Compared to participants aged 25 to 39 years, participants aged 18 to 24 years (aOR 2.8; 95% confidence interval (CI) 2.1–3.7), aged 40 to 59 years (aOR 1.3; CI 1.1–1.6) and those above 60 years old (aOR 3.9; CI 3.0-5.1) were at higher risk of screening delay. Furthermore, participants who were on ART for less than 6 months (aOR 1.6; CI 1.1–2.3) were at higher risk of screening delay, as compared to those on ART for more than 6 months. Finally, participants without a documented travel time to the clinic (aOR 1.7; CI 1.1–2.8) were at higher risk of screening delay as compared to those with travel time to the clinic of 60–120 min.

### Qualitative findings

A total of 16 female participants completed the in-depth qualitative interviews. It was later learned that three participants were not enrolled in the VITAL study. However, we retained the qualitative data from these participants with HIV in the study, though we do not present their sociodemographic data.

Participants’ ages ranged from 21 to 60 years (median = 38.3 [interquartile range: 32.6–44.8]), while most were between 26 and 46 years old (84.6%; *n* = 11). Almost all had some kind of education (primary education or above (69.2%; *n* = 9), other education (23.1%; *n* = 3)) and most were homeworkers (76.9%; *n* = 10). All were taking ART and most had a suppressed viral load (84.6%; *n* = 11). Ten (62.5%) had their last cervical cancer screening within the recommended timeframe, while four participants (25%) were delayed and two (12.5%) had never been screened.

**Fig. 2:**
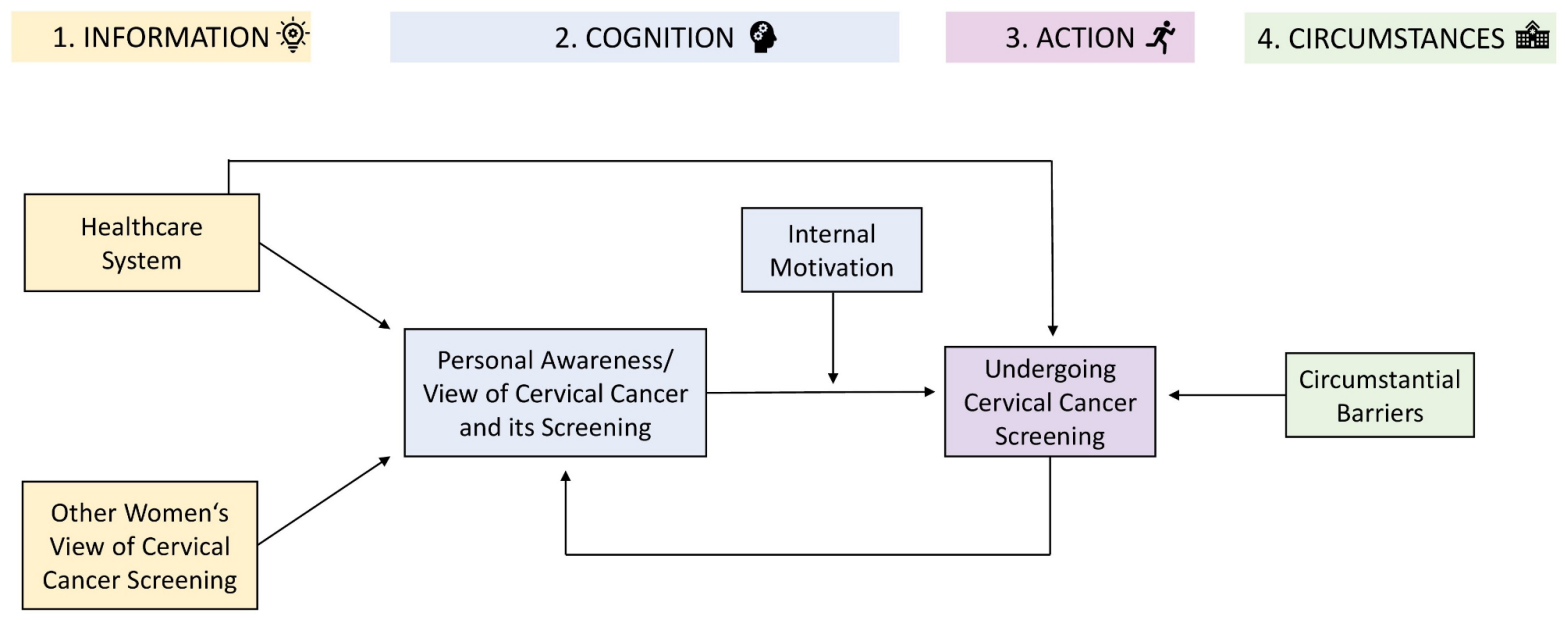
Pathway to cervical cancer screening

We identified four major themes in our qualitative analysis that comprised different steps leading to a decision to engage in cervical cancer screening for women in Lesotho: (1) provision of information and influence, (2) cognitive process, (3) action towards screening, and (4) circumstantial barriers. Figure 2 represents a model, developed for this analysis, showing the pathway to taking cervical cancer screening action as well as the interconnections between themes. Each theme is described below. The examination of qualitative results didn’t show any associations with age.

### Provision of information and influence

Women’s sources of information on cervical cancer screening came mainly from two sources: other women in the community and the local healthcare system. Participants mostly reported that the women they knew did not like cervical cancer screening, either because they were afraid of the process itself, viewed it as painful, or had heard that screening could lead to symptoms, such as longer menstrual cycles thereafter. Participants described that women in general were often misinformed about cervical cancer and its screening, which led to fear or dislike of it.

*“A lot of people are afraid to get tested for cancer because they say after testing for it*,* they experience their periods*,* which go on forever until the said person has to go to Maseru [capital city] because things don’t get better. That has instilled fear in them.” (ID#10*,* age 38*,* primary education*,* homeworker*,* last screening as per guidelines)*.

However, some participants reported that women they knew had positive feelings about cervical cancer screening because they cared about their health. Knowing they were cancer-free would bring them relief, and if cancer were detected, it could be treated, allowing for recovery.

*“They were happy because their results were negative. Even if they knew they were positive they would have cured it [cervical cancer] and left it behind.” (ID#15*,* last screening as per guidelines)*.

There were also a few participants who had not talked about this topic with other women and therefore did not know any other women who had undergone cervical cancer screening.

When it came to obtaining information from the healthcare system, participants emphasized the importance of raising awareness about cervical cancer as a serious disease, particularly for women with HIV, and communicating the importance and availability of cervical cancer screening. Given this, participants felt that the healthcare system, through encouragement by health workers, health campaigns, and screening reminders, was able to clear up some misconceptions about cervical cancer screening.

*“I haven’t heard in depth*,* but the health workers sometimes mention that [elevated cervical cancer risk in women with HIV] when they hold workshops and gatherings in the village. They hold gatherings at the royal grounds and inform us that women who are HIV positive are at a higher risk of having cervical cancer.” (ID#7*,* age 26*,* primary education*,* homeworker*,* last screening delayed)*.

Taken together, the two primary sources of information regarding cervical cancer screening came from other women and the healthcare system. Information from other women tended to be negative and fear-inducing, whereas information directly from the healthcare system was viewed as playing an important role to clarify misconceptions.

### Cognitive process on cervical cancer screening and taking action

Receiving information on cervical cancer and its screening represented the initial crucial step in the decision-making process for women regarding their own screening. Subsequently, women engaged in a cognitive process, gaining awareness and forming a view of cervical cancer screening. This ultimately impacted their decision to take action and undergo screening. Women were generally aware of the importance of cervical cancer screening when they shared their own opinions during the interview. Some saw the significance of cervical cancer as a serious disease and the importance of a timely diagnosis.

*“When it [cervical cancer] is discovered early*,* it is easy to cure it; but if it is discovered late*,* it intensifies and becomes difficult to manage and cure.” (ID#3*,* age 43*,* primary education*,* homeworker*,* last screening delayed)*.

However, many participants showed limited awareness of their increased personal risk of developing cervical cancer as women with HIV. Additionally, some were unaware of the recommended screening interval of every two years, and one participant was completely unaware of the option to undergo cervical cancer screening.

Some participants reported that anxiety about the screening procedure, based on reports from other women, was preventing women who with no cervical screening experience from getting screened. Several participants noted that their own negative expectations, influenced by these narratives, did not align with their own experience after they underwent the screening themselves. Specifically, these participants described their own screening experiences as rather positive and painless, in contrast to the prevailing negative perspective. This highlights the positive effect of having a prior cervical cancer screening experience to dissuade misconceptions.

*“When you have not experienced something*,* you will understand it to be as how other people say it is because you have not experienced it yourself. But when you do*,* you will realize that it was just an exaggeration*,* or that it is not like that at all.” (ID#3*,* age 43*,* primary education*,* homeworker*,* last screening delayed)*.

Nevertheless, one participant described her experience as painful, which reinforced her beliefs about the negative prevailing image of cervical cancer screening. However, because of her concern about her health and her awareness of its importance, she indicated pursuing screening nonetheless.

*“It was painful…that thing that is inserted in the private part. When you get home and urinate it becomes very painful…Honestly no [I wouldn’t get screened again] but I will do it for my health.” (ID#10*,* age 38*,* primary education*,* homeworker*,* last screening as per guidelines)*.

An important theme that emerged in the interviews was the presence or absence of internal motivation to undergo screening. Some women expressed their concern about their health, including their cervical cancer status, and as a result, pursued cervical cancer screening.

*“Every time I visited the facility like I did now*,* I made sure that I got tested. My opinion is that I have to be always informed about my health issues.” (ID#2*,* age 45*,* other education*,* employed/ self-employed*,* last screening as per guidelines)*.

At the same time, several women described not knowing why they had not screened for cervical cancer, despite their awareness of its importance and availability. They explained that they were not able to identify a motivating factor to undergo screening, which led some participants to conclude that they did not take cervical cancer screening seriously enough.

*“The health workers explain everything properly but I haven’t had anything in my mind that would make me want to visit the facility to get tested for cervical cancer…*.

*There is no reason for not getting tested*,* this is my health. It is simply the fact that as an individual there are certain things you don’t take seriously…Yes sir*,* in other words there are certain things one doesn’t take seriously for no reason at all and I would say I don’t take getting tested seriously.” (ID#7*,* age 26*,* primary education*,* homeworker*,* last screening delayed)*.

These same women further highlighted that the motivation needed to undergo cervical cancer screening must come from within the individual. During the discussion, these women stated the healthcare system would only be able to provide people with the necessary information, but that individuals needed to take responsibility for their own lives.

### Circumstantial barriers

There were two primary circumstantial barriers that participants described as hindering them from undergoing cervical cancer screening: personal factors and healthcare system factors. Personal factors were related to the timing of their menstrual cycle and having time to schedule a visit. In terms of the healthcare system, the primary issues were related to the sex of the provider and not being well enough informed on the circumstances of screening. Regarding provider sex, some women strongly preferred to undergo screening from a female healthcare provider because they felt more comfortable undressing in front of a woman. One participant also feared being sexually assaulted by a male healthcare provider, highlighting the importance of considering women’s personal preference when offering cervical cancer screening.

*“I am not able to be at ease with [a] male [provider]…it could be to rape me.” (ID#6*,* age 21*,* primary education*,* homeworker*,* never screened)*.

Finally, one woman described a scenario where she had been summoned to attend the clinic, but upon arrival, it was not clear what the purpose of the appointment was. She left the clinic without receiving services, only to find out later that she was supposed to have received cervical cancer screening at the appointment. This resulted in frustration and anger. This occurrence was further reinforced by another participant, stating issues with poor clinic operations as a reason that other women are reluctant to pursue screening.

## Discussion

This mixed-methods study sought to quantitatively characterize the demographic and clinical profile of women with HIV and delayed cervical cancer screening in Lesotho and understand possible reasons for delayed screening according to women’s own experiences. Overall, 47.9% of participants reported a delay in cervical cancer screening, including 40.5% who were never screened before. In the study, age, travel time to the clinic, and time on ART were associated with delayed cervical cancer screening. The qualitative data suggested a multi-step cognitive and behavioral process in women’s decision to undergo screening, with opportunities for intervention along the way.

The strongest effect size was found for participants’ age. The study’s finding that younger (18–24 years) and women above 40 (and especially above 60) were at higher risk of delay in screening stands in contrast to previous research conducted in South Africa [28] and Kenya [29]. Women with HIV who were approximately 35 years or older in South Africa and Kenya were actually more likely to screen for cervical cancer. Our findings regarding screening delay among women aged 18 to 24 could be explained by the screening onset at the age of 25 or when becoming sexually active, as recommended by the Lesotho screening guidelines. The delayed screening of this high-risk cervical cancer population implies that measures to support the uptake of screening are needed.

To understand these measures, our qualitative analysis identified four key themes related to the process of cervical cancer screening in women with HIV in Lesotho. One possible reason for the low uptake of this procedure was the negative influence of other women, who created fear and negative expectations about the cervical cancer screening process. This finding is echoed in a qualitative study of Zambian women, who perceived that women’s negative stories and related rumors of cervical cancer screening had a negative influence on the community’s perception of undertaking this procedure [30].

One unexpected factor that emerged from the qualitative data was related to women’s internal motivation (or lack thereof) when pursuing cervical cancer screening. Women described needing to identify a personal motivation to pursue screening; without this reason, simply knowing about the disease and screening availability was not enough for one to pursue screening. This suggests the need to possibly help women identify personal motivators to engage in cervical cancer screening. A recent mixed-methods study conducted among women with HIV in Ghana used the R3 model (reframe, reprioritize, reform) to give information, increase motivation, and develop a positive attitude towards cervical cancer screening [31]. The study found an increased cervical cancer screening rate compared to standard screening, and the intervention was perceived as acceptable, appropriate, and feasible among study participants. This model addresses important barriers identified in the current study related to lack of awareness, misconceptions, and low motivation, and could therefore be a promising strategy to evaluate in Lesotho.

Study findings must be interpreted in light of strengths and limitations. Strengths include the use of a mixed-methods design, a large sample size for the quantitative analysis, and data representative of women with HIV taking ART in care at rural health facilities in two districts of Lesotho. However, the exploration of cervical cancer screening within the qualitative interviews was limited by the interview guide covering multiple topics, which meant the time for the exploration on cervical cancer screening was limited. Furthermore, the three women from outside the defined VITAL study cohort were included in the qualitative interviews, but their demographic data were not available, leading us to characterize the qualitative sub-sample less well. The limited variability in age within the qualitative sub-sample may have contributed to not being able to identify any patterns in themes based on age. Finally, a few variables were not documented for a substantial fraction of our quantitative study population. For travel time to the clinic, those who had no reported data for this variable had a higher odds ratio for cervical screening delay, indicating that this result should be interpreted with caution. 

## Conclusion

This mixed-methods study indicates that 47.9% of women with HIV in Lesotho experience delayed cervical cancer screening and identified women with HIV in Lesotho under 24 years and over 40 years (and especially those over 60 years) to be at the greatest risk of having a delay in cervical cancer screening. Limited awareness of cervical cancer risks and screening guidelines, misconceptions and fears created through the narratives of other women, and low internal motivation appear to be major barriers to screening. These findings may help to tailor cervical cancer screening campaigns to overcome these barriers in Lesotho and similar settings.

## Electronic supplementary material

Below is the link to the electronic supplementary material.

Supplementary Material 1

## Data Availability

Data and materials are available upon reasonable request to the corresponding author.

## Abbreviations

ART: Antiretroviral therapy
HIV: Human immunodeficiency virus
HPV: Human papillomavirus
VITAL: Viral load triggered ART care in Lesotho
