# Supplementary material for: Cervical cancer screening delay and associated factors among women with HIV in Lesotho: a mixed-methods study
Source: BMC Womens Health. 2024 Oct 1;24:543. doi: 10.1186/s12905-024-03382-8 (PMC11446098; doi:10.1186/s12905-024-03382-8)
Supplement: Supplementary file 1 — Supplementary Material 1 [file 12905_2024_3382_MOESM1_ESM.pdf]

Supplementary material: Questions used to assess awareness, perception, barriers and facilitators to uptake of cervical cancer screening

1. *(For women only)* **You may know that women living with HIV are at greater risk for getting cervical cancer.**
    a. **When was the last time you had a cervical cancer screening?**
        i. ***Probe:*** How often have you gotten screened in the last 5 years?

    b. *(screened previously)* **What led to you getting screened the last time? For example, did your healthcare provider recommend it or did you need to request this service? Please explain.**
        i. ***Probe for those who screened less than every 2 years:*** What are your thoughts on being screened more often? Please explain.

    c. *(never screened)* **Tell me why you never received a screening for cervical cancer.**
        i. ***Probe:*** What do you think about cervical cancer screening?
        ii. ***Probe:*** Would you take the cervical cancer screening if offered, why or why not?
        iii. ***Probe:*** Would it matter if it was offered by a male or female provider? Please explain.

    d. **Do you know other women in your life who have been screened for cervical cancer?**
        i. **Probe:** Who was it and what was/were their experience(s)?

    e. **What is the general perception of cervical cancer screening in the community for women?**
